# Family analysis and literature study of hereditary hypophosphatemic rickets with hypercalciuria

**DOI:** 10.1186/s12887-024-04589-2

**Published:** 2024-02-14

**Authors:** Lufeng Wang, Gulimire Kulaixi, Jiazireya Zaiyinati, Guzhalikezi Aibai, Danyang Du, Yanying Guo

**Affiliations:** 1 Department of Endocrinology, People′s Hospital of Xinjiang Uygur Autonomous Region, Xinjiang Clinical Research Center for Diabetes, Urumqi, 830000 China; 2 Department of Endocrinology, Yecheng County, Kashi City of Xinjiang Uygur Autonomous Region, Kashi City, 832000 China

**Keywords:** HHRH, *SLC34A3*, Hypophosphatemia, Nephrocalcinosis, Hypercalciuria

## Abstract

**Background:**

Hereditary hypophosphatemia rickets with hypercalciuria (HHRH) is a rare autosomal recessive disorder characterised by reduced renal phosphate reabsorption leading to hypophosphataemia, rickets and bone pain. Here, we present a case of HHRH in a Chinese boy.

**Case presentation:**

We report a 11-year-old female proband, who was admitted to our hospital with bilateral genuvarum deformity and short stature. Computed Tomography (CT) showed kidney stones, blood tests showed hypophosphatemia, For a clear diagnosis, we employed high-throughput sequencing technology to screen for variants. Our gene sequencing approach encompassed whole exome sequencing, detection of exon and intron junction regions, and examination of a 20 bp region of adjacent introns. Flanking sequences are defined as ±50 bp upstream and downstream of the 5′ and 3′ ends of the coding region.The raw sequence data were compared to the known gene sequence data in publicly available sequence data bases using Burrows-Wheeler Aligner software (BWA, 0.7.12-r1039), and the pathogenic variant sites were annotated using Annovar. Subsequently, the suspected pathogenic variants were classified according to ACMG’s gene variation classification system. Simultaneously, unreported or clinically ambiguous pathogenic variants were predicted and annotated based on population databases. Any suspected pathogenic variants identified through this analysis were then validated using Sanger sequencing technology. At last, the proband and her affected sister carried pathogenic homozygous variant in the gene*SLC34A3*(exon 13, c.1402C > T; p.R468W). Their parents were both heterozygous carriers of the variant. Genetic testing revealed that the patient has an*LRP5*(exon 18, c.3917C > T; p.A1306V) variant of Uncertain significance, which is a rare homozygous variant.

**Conclusion:**

This case report aims to raise awareness of the presenting characteristics of HHRH. The paper describes a unique case involving variants in both the*SLC34A3*and*LRP5*genes, which are inherited in an autosomal recessive manner. This combination of gene variants has not been previously reported in the literature. It is uncertain whether the presence of these two mutated genes in the same individual will result in more severe clinical symptoms. This report shows that an accurate diagnosis is critical, and with early diagnosis and correct treatment, patients will have a better prognosis.

**Supplementary Information:**

The online version contains supplementary material available at10.1186/s12887-024-04589-2.

## Introduction

Hypophosphatemic rickets (HR), also known as familial hypophosphatemia, is characterized by hypophosphatemia and phosphate loss in the kidney. It is mainly a genetic disorder of bone mineralization [1]. It includes a group of diseases with similar phenotypes but different genotypes, patterns of inheritance and aetiology, the most common being X-linked dominant hypophosphatemic rickets. Less common may be caused by autosomal dominant or recessive inheritance [2]. Hereditary hypophosphatemia rickets with hypercalciuria (HHRH) is an autosomal recessive metabolic disorder of childhood, in which loss of NPT2c dependent phosphate transport in the renal proximal tubule leads to hypophosphatemic rickets and/or osteomalacia [3]. Some studies showed that human NPT2c is N-glycosylated in the ER and then undergoes further modification upon appropriate folding and passing the ER quality control system, which is followed by apical expression. The protein misfolding may be caused by the p.R468W variant [4]. It characterized by reduced renal phosphate reabsorption, hypophosphatemia and rickets. It differs from other forms of hypophosphatemia in that elevated serum levels of 1, 25-dihydroxyvitamin D led to hypercalciuria. Osteoporosis-pseudoglioma syndrome (OPPG; OMIM #259770) is less common disease, this disease is related to the *LRP5*. Typical characteristics of OPPG are early onset (juvenile) osteoporosis associated with ocular abnormalities involving vitreoretinal structures (pseudoglioma) that can lead to blindness [5]. Currently, burosumab has been approved for the treatment of children and adults with X-linked hypophosphatemia (XLH), data from phase 2 and 3 trials report overall safety and efficacy. However, in patients with HHRH, there have been no studies confirming the efficacy and prognosis of this drug. To arouse attention to these two diseases and reduce the occurrence of sequelae, the clinical manifestations, neuroimaging characteristics, diagnosis and treatment of 1 case of HHRH and OPPG admitted to our hospital were reported and a literature study was performed.

## Case presentation

The patient, an 11-year-old female, has been suffering of short stature for 6 years before being admitted to the hospital. The girl was the fifth child born to a nonconsanguineous couple. She was born naturally at full term, with a low birth weight and height, and was breastfed. After the age of 5, the patient’s height is lower than that of children of the same age. After admission to our hospital, Blood tests showed the decreased levels of phosphorus to 0.80 mmol/L (normal range: 0.96–1.62 mmol/L), 1,25-(OH)2-D to 11.39 pg/ml (normal range: 20–100 pg/ml), tubular reabsorption of phosphate (TRP) to 83.7% (normal range: 84 to 96%) and increased levels of alkaline phosphatase to 1427.40 U/L (normal range: 45–125 U/L). The other electrolytes, thyroid hormone, 24-hour urine calcium levels and ratio of maximum rate of renal tubular reabsorption of phosphate to glomerular filtration rate (TMP/GFR) were unremarkable. The patient’s siblings and parents blood phosphorus results revealed that one of the patient’s sisters had low blood phosphorus, and bowed legs were the only clinical manifestation. Here are the patient’s laboratory test results along with their corresponding normal reference ranges.

Table 1 Phosphate clearance test results: after consuming 300 ml of distilled water on an empty stomach, the following measurements were taken 2 hours later; TRP: tubular reabsorption of phosphate; TMP/GFR: ratio of maximum rate of renal tubular reabsorption of phosphate to glomerular filtration rate.

**Table 1:** Phosphate clearance test results

Item	creatinine	phosphorus	TRP	TMP/GFR
blood	20.86 umol/L	0.99 mmol/L	83.7% (84 to 96%)	0.829 mmol/l (0.8–1.35 mmol/l)
urine	1297.9 umol/L	10.02 mmol/L		

**Fig. 1:**
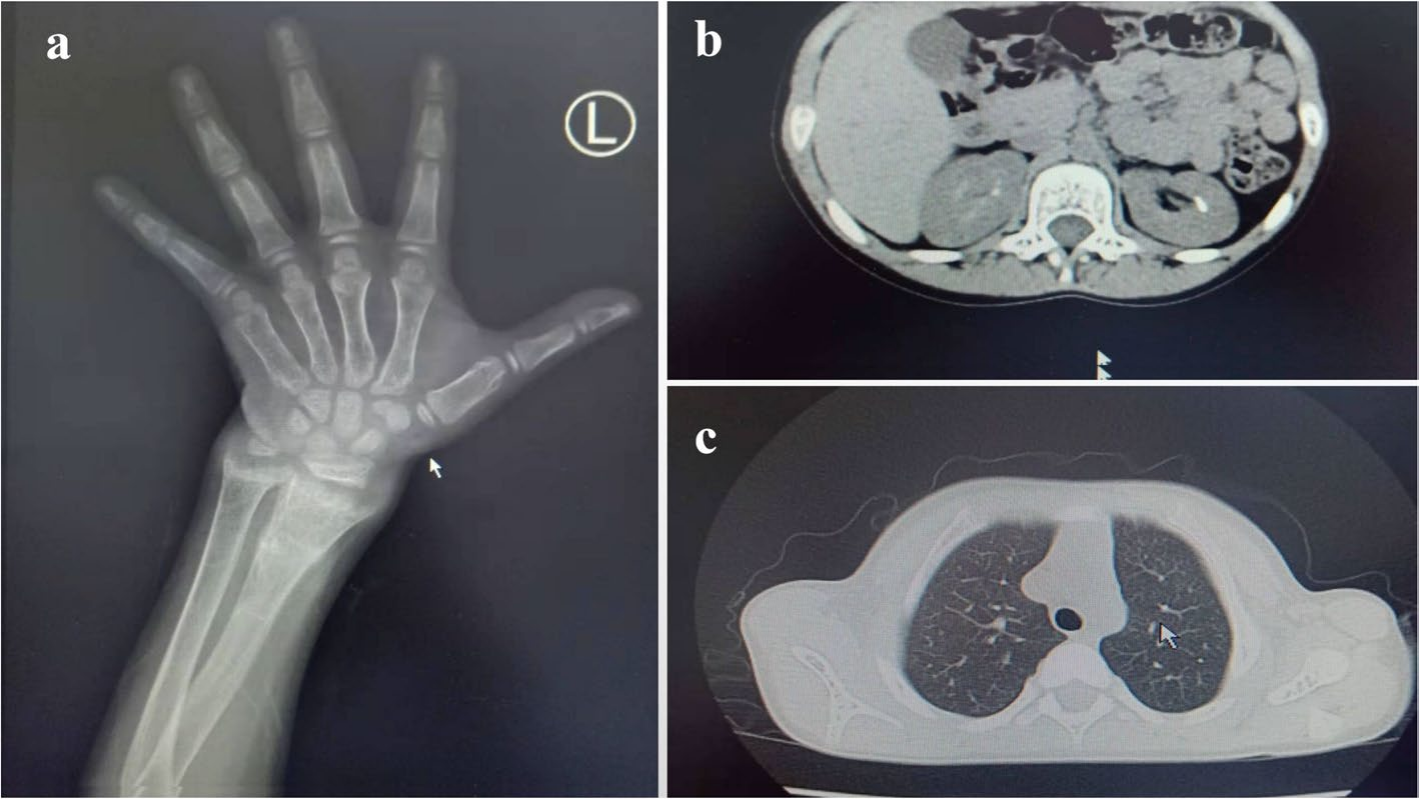
A skeletal survey of the patient.**a**Widening of the distal ulnar radius epiphysis on the left side, spreading to both sides, central depression, cup-shaped changes, irregular brush-like cup, widening of the epiphyseal plate, jagged faint translucent line shadow is seen on the distal radius on the left side.**b**A strong echogenic cluster of about 6 mm in the right renal pelvis and calyces.**c**Cup-like depression of the sternal segments of the ribs with a brush-like appearance

Hand X-ray showed left distal ulnar radius consistent with rickets in active phase (Fig.1a), radiographs of growth plates demonstrate metaphyseal widening, cupping, lucency and flaring, possible old fracture of the distal radius on the left side, and bone age comparable to the girl’s standard of 9 years; renal scan suggested a strong echogenic cluster of about 6 mm in the right renal pelvis and calyces (Fig.1b); the chest radiograph showed reduced bone density in the bones within the scan area (Fig.1c); previous bone X-rays of both hands suggested that the hands and wrist joints were dysplastic and rickets was considered; radiographs of both lower limbs suggested that rickets was present in both lower limbs with bowed legs. Pure tone audiometry: the average hearing threshold at speech frequencies of 20 dB in both ears. The conductance map shows a binaural (type A) curve, no otoacoustic emissions elicited in either ear. Auditory brainstem: binaural hearing thresholds of 25dBnHL, suggestive of normal hearing.

**Fig. 2:**
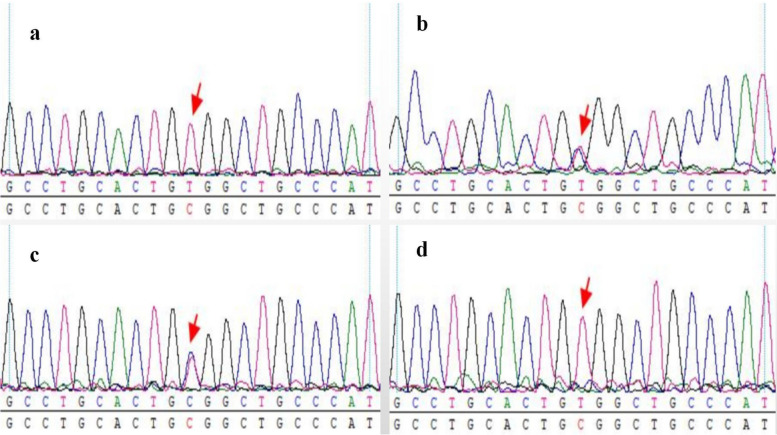
Results of Sanger sequencing for patient.**a**The proband carry a homozygous disease-causing variant in the gene*SLC34A3*(exon 13, c.1402C>T; p.R468W),**b**The proband’s father carry a heterozygous variant of the gene,**c**The proband’s mother carry a heterozygous variant of the gene,**d**the proband’s sister has a homozygous variant

**Fig. 3:**
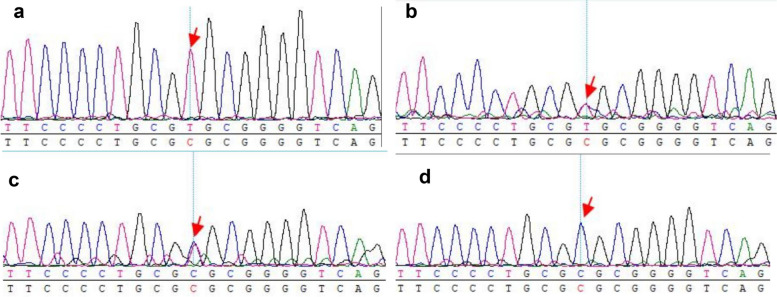
Results of Sanger sequencing for patient.**a**The proband carry a homozygous variant in the gene*LRP5*(exon 18 c.3917CT; p.A1306V),**b**The proband’s father carry a heterozygous variant of the gene,**c**The proband’s mother carry a heterozygous variant of the gene,**d**the proband’s sister has not variant

A case is reported in this paper. The proband’s parents are not consanguineous. With the informed consent of the patients and family members, whole exome sequencing analysis was performed and showed that the proband harbored the c.1402C > T; p.R468W in the*SLC34A3*gene (Fig.2a), this variant has been described by Bergwitz et al. 2006 [6]. Sanger sequencing was performed to verify this variant in other family members. The proband’s mother (Fig.2c) and father (Fig.2b) carry a heterozygous variant of the gene, and the proband’s sister (Fig.2d) had a homozygous variant. The proband also harbored a c.3917C > T (p.A1306V) homozygous variant in the*LRP5*gene (Fig.3a). The proband’s mother (Fig.3c) and father (Fig.3b) carried a heterozygous variant in the gene, but the proband’s sister (Fig.3d) was normal.

Therefore, combined with the clinical manifestations of the patient, the genetic testing results and family analysis can be used to diagnose hereditary hypophosphatemic rickets with hypercalciuria. The relevant manifestations of the patient and her relatives are as follows (Table 2).

**Table 2:** Comparison of clinical symptoms

	SLC34A3	LRP5	Clinical Characteristics	
Father	Heterozygous variant	Heterozygous variant	Height: 175 cm	Normal blood phosphorus
Mother	Heterozygous variant	Heterozygous variant	Height: 160 cm	
Sister	Homozygous variant	Not variant	Height: 158 cm Age: 13 years old	Hypophosphatemic and lower-extremity bowing
The proband	Homozygous variant	Homozygous variant	Height: 104 cm Age: 11 years old	Hypophosphatemia, renal calculi, the TRP were slightly lower than normal, metaphyseal widening, cupping, flaring, lucency, lower-extremity bowing and short stature

## Discussion

HHRH is a rare metabolic disorder with an autosomal recessive inheritance pattern. Tieder et al. were the first to describe the clinical phenotype of HHRH in a consanguineous Bedouin family [7]. HHRH is characterized by hypophosphatemia, short stature, rickets and/or osteomalacia, and secondary absorptive hypercalciuria. Hypophosphatemia stimulates renal 1-alpha-hydroxylase, which increases 1,25-dihydroxyvitamin D synthesis. This results in the suppression of parathyroid hormone (PTH) and increases the intestinal absorption of calcium, which increases the renal filtered calcium load and hypercalciuria [8, 9]. This helps differentiate HHRH from autosomal dominant hypophosphatemic rickets (ADHR) and X-linked hypophosphatemia (XLH). A number of genes associated with skeletal or growth abnormalities have been identified (Table 3), among which more than 9,000 pathogenic variants were found in these genes (Table 4). Please refer to the supplementary material for details (Additional file 3). These genes can help us to better understand such disorders. In this case, the main clinical manifestations were bilateral genu varum deformity and short stature. However, the patient did not present with bone pain, and investigations suggested hypophosphatemia, rickets and renal calculi, the TRP were slightly lower than normal. Therefore, gene sequencing was performed to determine the cause.

Bergwitz et al. [6] performed a genome-wide linkage scan combined with homozygosity mapping in the large consanguineous Bedouin kindred reported in Tieder et al. [7]. The candidate gene region was narrowed down to the 1.6 MB region on chromosome 9q34, and the mutated gene was identified as*SLC34A3*. This gene encodes the type 2C sodium phosphate cotransport protein (NPT2c), which, together with NPT2a, is expressed in the apical region of proximal renal tubule cells under the control of PTH and fibroblast growth factor 23(FGF23). Additionally, phosphate is reabsorbed from the glomerular filtrate [10, 11].

Clinically, HHRH patients with homozygous or compound heterozygous*SLC34A3*/NPT2c variants usually present with hypophosphatemia, rickets, or osteomalacia after reduced renal phosphate reabsorption and often develop kidney stones or renal calcification. In contrast,*LRP5*gene (c.C3917T, p. A1306V) variant was found in the proband by genetic testing, and according to the ACMG guidelines [12], this variation was preliminarily determined to be of uncertain significance, the evidence supporting its pathogenicity is insufficient, but the association with the clinical phenotype of OPPG may exist. OPPG is caused by biallelic variants in the gene encoding low-density lipoprotein receptor-related protein 5 (*LRP5*) [13], which often presents with vitreoretinal degeneration such as microphthalmia, phthisis bulbi, retinal detachments, persistent fetal vasculature, congenital retinal folds, exudative retinopathy, cataracts, and retinal calcifications. Skeletal abnormalities result from reduced bone mass and usually present as recurrent long bone and vertebral fractures, compressed vertebrae, bowing of long bones, short stature, and kyphosis [5]. Although the patient in this case presented with some of the clinical manifestations described above, a different outcome with lower vitamin D levels was also seen. The reasons considered may be related to the high latitude, the spring visit and the relatively low vitamin D production due to reduced activity during school age. There are no clinical data to show the average vitamin D levels in children under the same conditions, and further exploration of the possible causes is needed.

Since HHRH is an autosomal recessive genetic disease, the typical manifestations of the disease require double-allelic variants. The loss of just one*SLC34A3*allele does not necessarily lead to abnormal laboratory results. Therefore, the patient must inherit the variant from both parents to obtain the complete presentation of the disease. In one study [14], heterozygous patients presented with mild clinical symptoms, usually mild or absent hypophosphatemia, and no skeletal deformities, with recurrent kidney stones and hypercalciuria as the main manifestations compared with homozygous patients. In homozygous patients, hypophosphatemia and kidney stones are more common. In this case report, the patient had a homozygous gene variant, and the patient had hypophosphatemia, rickets, short stature, and kidney stones, which was consistent with the report. However, the*LRP5*gene also is mutated in the patient. The disorder is called osteoporosis-pseudoglioma syndrome and causes short stature in patients with early onset of clinical symptoms. The variation has been preliminarily determined to be of uncertain significance, and the evidence supporting its pathogenicity is insufficient, further exploration is needed to determine whether short stature is derived from this gene pathogenic variant, and whether these two genetic variants lead to more severe clinical manifestations when present together in the same individual.

A review of the patient’s siblings and parents blood phosphorus results revealed that one of the patient’s sisters had low blood phosphorus, and bowed legs were the only clinical manifestation. The reason for this may be related to the proband having two homozygous variants in the*SLC34A3*and*LRP5*genes, whereas only the homozygous variant in the*SLC34A3*gene was present in the sister. Subsequently, genetic testing of the parents of the proband and the sister who showed only hypophosphatemia on laboratory tests confirmed the presence of a heterozygous variant in the gene in the affected parents and a homozygous gene variant in the sister. A recent study analysed the effect of the*SLC34A3*variant on NPT2c function and the link with the phenotype of the patients. In the end, no association was found between the severity of the disease and the degree of phosphate transport impairment [15]. However, the variant was not analyzed in the above articles. Therefore, whether there is clinical heterogeneity in the case for the homozygous gene variant c.1402C > T; p.R468W in the*SLC34A3*gene warrants further investigation.

HHRH is easily overlooked or underdiagnosed. Genetic evaluation of patients with familial hypercalciuria, hypophosphatemia and kidney stones is needed to further understand the prevalence and treatment of this rare disease. Biochemical, renal ultrasound and genetic screening should also be considered for first-degree relatives of patients with HHRH to increase confidence in the diagnosis of the disease.

Early and accurate diagnosis of HHRH has important therapeutic significance. Tinder et al. [16] emphasized the importance of correct diagnosis, as long-term phosphate therapy alone can reverse clinical and biochemical abnormalities of HHRH. However, treatment with active vitamin D metabolites may aggravate the disease, leading to hypercalcemia, nephrocalcinosis, and renal damage. Blood phosphorus levels are restored to nearly normal levels by phosphorus supplementation, which is conducive to fracture healing, and phosphorus supplementation can reduce blood calcium. A study [17] reported a 14-year-old Turkish boy with severe rickets since age 2 years. Laboratory analysis showed hypophosphatemia, normocalcemia, increased alkaline phosphatase activity, and normal serum PTH. Serum 1,25(OH)2D3 was markedly increased. Trabecular bone biopsy showed osteomalacia. At 10 years of age, high doses of vitamin D (40.000 U/D) and phosphate (1.1 g/D) for 20 days resulted in symptomatic kidney stones.

In one case report [18], genetic testing was performed on a 32-year-old woman with a painful valgus knee deformity and after a definitive diagnosis of HHRH, she was started on phosphate supplementation and suspended vitamin D. She subsequently showed significant improvement in her left knee pain. Six months after starting phosphate supplementation, she underwent a simple left total knee arthroplasty (TKA), which significantly improved the patient’s quality of life after the procedure. In contrast, this paper proposes that corrective surgery (e.g. osteotomy and TKA) can be performed in patients presenting with limb deformities without timely and definitive diagnosis and intervention for HHRH and that phosphate supplementation should be administered for at least 6 months prior to surgery to ensure that bone mineralization does not continue to be compromised. Otherwise, poor bone healing and mineralization may occur, leading to further irreversible defects [17].

Treatment of HHRH consists of monotherapy with oral Pi supplements, while active vitamin D analogs are contraindicated. This is mainly because endogenous 1,25(OH)2D levels are already elevated but also to prevent further worsening of hypercalciuria [19]. Burosumab, a human monoclonal anti-FGF23 antibody, was recently approved as a specific therapy for XLH, data from phase 2 and 3 trials report overall safety and efficacy. However, in patients with HHRH, there have been no studies confirming the efficacy and prognosis of this drug. Further research and clinical trials are needed to verify the therapeutic effect and prognosis of the drug on HHRH patients. Overall, it is not clear which genetic and biochemical indicators best predict the risk of renal calcification in the HHRH family and whether oral phosphate supplements alone are safe for long-term treatment in terms of renal calcification. It is also not clear whether renal phosphate leakage will last for life and whether treatment can be stopped after long-term treatment. Therefore, there is a large amount of data needed to support how oral phosphate therapy should be monitored.

## Conclusion

This paper describes a case of*SLC34A3*and*LRP5*gene variants inherited in an autosomal recessive manner. The*SLC34A3*gene variant is considered a mutant gene for HHRH. The*LRP5*gene variant is considered a gene of uncertain significance and has hitherto not been described in the context of the two genes at the same time. It is inconclusive whether the superposition of the two gene variants exacerbates clinical symptoms. This report shows that an accurate diagnosis is critical, and with early diagnosis and correct treatment, patients will have a better prognosis.

### Supplementary Information

**Additional file 1.**

**Additional file 2.**

**Additional file 3.**

## Data Availability

The*SLC34A3*genes analyse in the current study are available in the dbSNP repository, rs121918238, The*LRP5*genes analyse in the current study are available in the dbSNP repository, rs772148405https://www.ncbi.nlm.nih.gov/snp/.
