# Supplementary material for: Family analysis and literature study of hereditary hypophosphatemic rickets with hypercalciuria
Source: BMC Pediatr. 2024 Feb 14;24:121. doi: 10.1186/s12887-024-04589-2 (PMC10865686; doi:10.1186/s12887-024-04589-2)
Supplement: Supplementary file 1 — Additional file 1. [file 12887_2024_4589_MOESM1_ESM.pdf]

Table 2. Other Genetic of Renal Phosphate Wasting.

Phenotype	Phenotype	Clinical Characteristics
AD hypophosphatemic rickets (ADHR)	FGF23	Renal phosphate wasting w/o hypercalciuria
AR hypophosphatemic rickets	DMP1<br>ENPP1	Renal phosphate wasting w/o hypercalciuria
McCune-Albright syndrome	GNAS	Hypophosphatemic rickets
Cutaneous skeletal hypophosphatemia syndrome	HRAS<br>KRAS<br>NRAS	Hypophosphatemia is frequent & biochemically indistinguishable from that seen in XLH.
Hereditary hypophosphatemic rickets with hypercalciuria	*SLC34A3*	Hypophosphatemia; hypercalciuria
Hypophosphatemic nephrolithiasis/osteoporosis	*SLC34A1*<br>*SLC9A3R1*	Hypophosphatemia; hypercalciuria
Hypophosphatemic rickets, X-linked recessive	*CLCN5*	Hypophosphatemia; hypercalciuria
Raine syndrome	*FAM20C*	Osteosclerotic skeletal changes; hypophosphatemia
Osteoglophonic dysplasia	*FGFR1*	Hypophosphatemia; lower than expected calcitriol levels
Hypophosphatemia rickets with hyperparathyroidism	*KL*	Hypophosphatemia; inappropriately normal calcitriol level
