# Supplementary material for: Family analysis and literature study of hereditary hypophosphatemic rickets with hypercalciuria
Source: BMC Pediatr. 2024 Feb 14;24:121. doi: 10.1186/s12887-024-04589-2 (PMC10865686; doi:10.1186/s12887-024-04589-2)
Supplement: Supplementary file 2 — Additional file 2. [file 12887_2024_4589_MOESM2_ESM.docx]

Table 2. The variants in hereditary hypophosphatemic rickets with hypercalciuria.

| Gene(s) | Variation  Location | Protein change | Clinical significance |
| --- | --- | --- | --- |
| *SLC34A3* | [NM_001177316.2(SLC34A3): c.176-9T>G](https://www.ncbi.nlm.nih.gov/clinvar/variation/2436020/)  GRCh37: Chr9: 140127018  GRCh38: Chr9: 137232566 |  | Uncertain significance |
| *SLC34A3* | [NM_001177316.2(SLC34A3): c.847-26T>C](https://www.ncbi.nlm.nih.gov/clinvar/variation/2436019/)  GRCh37: Chr9: 140128289  GRCh38: Chr9: 137233837 |  | Uncertain significance |
| *SLC34A3* | [NM_001177316.2(SLC34A3): c.214_216del (p.Ser72del)](https://www.ncbi.nlm.nih.gov/clinvar/variation/2436017/)  GRCh37: Chr9: 140127063-140127065  GRCh38: Chr9: 137232611-137232613 | S72del | Uncertain significance |
| *SLC34A3* | [NM_001177316.2(SLC34A3): c.1783G>C (p.Ala595Pro)](https://www.ncbi.nlm.nih.gov/clinvar/variation/2234621/)  GRCh37: Chr9: 140130851  GRCh38: Chr9: 137236399 | A595P | Uncertain significance |
| *SLC34A3* | [NM_001177316.2(SLC34A3): c.1776G>C (p.Glu592Asp)](https://www.ncbi.nlm.nih.gov/clinvar/variation/1946490/)  GRCh37: Chr9: 140130844  GRCh38: Chr9: 137236392 | E592D | Uncertain significance |
| *SLC34A3* | [NM_001177316.2(SLC34A3): c.1796T>A (p.Leu599Ter)](https://www.ncbi.nlm.nih.gov/clinvar/variation/1705377/)  GRCh37: Chr9: 140130864  GRCh38: Chr9: 137236412 | L599* | Uncertain significance |
| *SLC34A3* | [NM_001177316.2(SLC34A3): c.*14A>T](https://www.ncbi.nlm.nih.gov/clinvar/variation/1705043/)  GRCh37: Chr9: 140130882  GRCh38: Chr9: 137236430 |  | Benign |
| *SLC34A3* | [NM_001177316.2(SLC34A3): c.1764C>T (p.Tyr588=)](https://www.ncbi.nlm.nih.gov/clinvar/variation/1650917/)  GRCh37: Chr9: 140130832  GRCh38: Chr9: 137236380 |  | Likely benign |
| *SLC34A3* | [NM_001177316.2(SLC34A3): c.1753G>A (p.Ala585Thr)](https://www.ncbi.nlm.nih.gov/clinvar/variation/1443292/)  GRCh37: Chr9: 140130821  GRCh38: Chr9: 137236369 | A585T | Uncertain significance |
| *SLC34A3* | [NM_001177316.2(SLC34A3): c.1785dup (p.Ser596fs)](https://www.ncbi.nlm.nih.gov/clinvar/variation/1318766/)  *GRCh37:* Chr9: 140130851-140130852  *GRCh38:* Chr9: 137236399-137236400 | S596fs | Uncertain significance |
| *SLC34A3* | [NM_001177316.2(SLC34A3): c.1242C>A (p.Tyr414Ter)](https://www.ncbi.nlm.nih.gov/clinvar/variation/1179153/)  *GRCh37:* Chr9: 140129090  *GRCh38:* Chr9: 137234638 | Y414* | Likely pathogenic |
| *SLC34A3* | [NM_001177316.2(SLC34A3): c.1795T>C (p.Leu599=)](https://www.ncbi.nlm.nih.gov/clinvar/variation/1168370/)  *GRCh37:* Chr9: 140130863  *GRCh38:* Chr9: 137236411 |  | Benign |
| *SLC34A3* | [NM_001177316.2(SLC34A3): c.1779C>G (p.Ile593Met)](https://www.ncbi.nlm.nih.gov/clinvar/variation/1167719/)  *GRCh37:* Chr9: 140130847  *GRCh38:* Chr9: 137236395 | I593M | Benign |
| *SLC34A3* | [NM_001177316.2(SLC34A3): c.1783G>A (p.Ala595Thr)](https://www.ncbi.nlm.nih.gov/clinvar/variation/1036876/)  *GRCh37:* Chr9: 140130851  *GRCh38:* Chr9: 137236399 | A595T | Uncertain significance |
| *SLC34A3* | [NM_001177316.2(SLC34A3): c.980T>C (p.Leu327Pro)](https://www.ncbi.nlm.nih.gov/clinvar/variation/930232/)  *GRCh37:* Chr9: 140128615  *GRCh38:* Chr9: 137234163 | L327P | Uncertain significance |
| *SLC34A3* | [NM_001177316.2(SLC34A3): c.85G>A (p.Gly29Arg)](https://www.ncbi.nlm.nih.gov/clinvar/variation/810483/)  *GRCh37:* Chr9: 140126239  *GRCh38:* Chr9: 137231787 | G29R | Uncertain significance |
| *SLC34A3* | [NM_001177316.2(SLC34A3): c.944del (p.Gly315fs)](https://www.ncbi.nlm.nih.gov/clinvar/variation/591424/)  *GRCh37:* Chr9: 140128577  *GRCh38:* Chr9: 137234125 | G315fs | Uncertain significance |
| *SLC34A3* | [NM_001177316.2(SLC34A3): c.1601_1633dup (p.Leu534_Val544dup)](https://www.ncbi.nlm.nih.gov/clinvar/variation/591121/)  *GRCh37:* Chr9: 140130662-140130663  *GRCh38:* Chr9: 137236210-137236211 |  | Uncertain significance |
| *SLC34A3* | [NM_001177316.2(SLC34A3): c.1765G>A (p.Glu589Lys)](https://www.ncbi.nlm.nih.gov/clinvar/variation/452005/)  *GRCh37:* Chr9: 140130833  *GRCh38:* Chr9: 137236381 | E589K | Uncertain significance |
| *SLC34A3* | [NM_001177316.2(SLC34A3): c.*14A>C](https://www.ncbi.nlm.nih.gov/clinvar/variation/194276/)  *GRCh37:* Chr9: 140130882  *GRCh38:* Chr9: 137236430 |  | Benign |
| *SLC34A3* | [NM_001177316.2(SLC34A3): c.585C>T (p.His195=)](https://www.ncbi.nlm.nih.gov/clinvar/variation/64505/)  *GRCh37:* Chr9: 140127685  *GRCh38:* Chr9: 137233233 |  | Uncertain significance |
| *SLC34A3* | [NM_001177316.2(SLC34A3): c.572G>A (p.Gly191Asp)](https://www.ncbi.nlm.nih.gov/clinvar/variation/64504/)  *GRCh37:* Chr9: 140127672  *GRCh38:* Chr9: 137233220 | G191D | Uncertain significance |
| *SLC34A3* | [NM_001177316.2(SLC34A3): c.516C>G (p.Thr172=)](https://www.ncbi.nlm.nih.gov/clinvar/variation/64503/)  *GRCh37:* Chr9: 140127523  *GRCh38:* Chr9: 137233071 |  | Uncertain significance |
| *SLC34A3* | [NM_001177316.2(SLC34A3): c.472C>T (p.Pro158Ser)](https://www.ncbi.nlm.nih.gov/clinvar/variation/64502/)  *GRCh37:* Chr9: 140127479  *GRCh38:* Chr9: 137233027 | P158S | Uncertain significance |
| *SLC34A3* | [NM_001177316.2(SLC34A3): c.439G>A (p.Ala147Thr)](https://www.ncbi.nlm.nih.gov/clinvar/variation/64501/)  *GRCh37:* Chr9: 140127370  *GRCh38:* Chr9: 137232918 | A147T | Uncertain significance |
| *SLC34A3* | [NM_001177316.2(SLC34A3): c.245G>T (p.Ser82Ile)](https://www.ncbi.nlm.nih.gov/clinvar/variation/64500/)  *GRCh37:* Chr9: 140127096  *GRCh38:* Chr9: 137232644 | S82I | Uncertain significance |
| *SLC34A3* | [NM_001177316.2(SLC34A3): c.218T>C (p.Val73Ala)](https://www.ncbi.nlm.nih.gov/clinvar/variation/64499/)  *GRCh37:* Chr9: 140127069  *GRCh38:* Chr9: 137232617 | V73A | Uncertain significance |
| *SLC34A3* | [NM_001177316.2(SLC34A3): c.1051C>T (p.Arg351Cys)](https://www.ncbi.nlm.nih.gov/clinvar/variation/64497/)  *GRCh37:* Chr9: 140128686  *GRCh38:* Chr9: 137234234 | R351C | Uncertain significance |

For more information, please visit the website: <https://maayanlab.cloud/Harmonizome/resource/ClinVar>

| Phenotype | Phenotype | Clinical Characteristics |  |
| --- | --- | --- | --- |
| AD hypophosphatemic rickets (ADHR) | FGF23 | Renal phosphate wasting w/o hypercalciuria |  |
| AR hypophosphatemic rickets | DMP1  ENPP1 | Renal phosphate wasting w/o hypercalciuria |  |
| [McCune-Albright syndrome](https://www.ncbi.nlm.nih.gov/books/n/gene/mccune-albright/) | GNAS | Hypophosphatemic rickets |  |
| Cutaneous skeletal hypophosphatemia syndrome | HRAS KRAS NRAS | Hypophosphatemia is frequent & biochemically indistinguishable from that seen in XLH. |  |
| Hereditary hypophosphatemic rickets with hypercalciuria | *SLC34A3* | Hypophosphatemia; hypercalciuria |  |
| Hypophosphatemic nephrolithiasis/osteoporosis | *SLC34A1* *SLC9A3R1* | Hypophosphatemia; hypercalciuria |  |
| Hypophosphatemic rickets, [X-linked recessive](https://www.ncbi.nlm.nih.gov/books/n/gene/glossary/def-item/x-linked-recessive/) | *CLCN5* | Hypophosphatemia; hypercalciuria |  |
| Raine syndrome | *FAM20C* | Osteosclerotic skeletal changes; hypophosphatemia |  |
| Osteoglophonic dysplasia | *FGFR1* | Hypophosphatemia; lower than expected calcitriol levels |  |
| Hypophosphatemia rickets with hyperparathyroidism | *KL* | Hypophosphatemia; inappropriately normal calcitriol level |  |
