# Supplementary material for: Family analysis and literature study of hereditary hypophosphatemic rickets with hypercalciuria
Source: BMC Pediatr. 2024 Feb 14;24:121. doi: 10.1186/s12887-024-04589-2 (PMC10865686; doi:10.1186/s12887-024-04589-2)
Supplement: Supplementary file 3 — Additional file 3. [file 12887_2024_4589_MOESM3_ESM.docx]

Table 3. List of 241 abnormal skeletal/growth genes identified.

| *ACAN* | *COL9A3* | *GJA1* | *NEU1* | *SIL1* |
| --- | --- | --- | --- | --- |
| *ADAMTS10* | *COMP* | *GLB1* | *NF1* | *SLC26A2* |
| *ADAMTS2* | *CRTAP* | *GLI3* | *NIPBL* | *SLC29A3* |
| *ADAMTSL2* | *CTDP1* | *GNAS* | *NOG* | *SLC2A2* |
| *AGPS* | *CTSK* | *GNPAT* | *NPR2* | *SLC34A3* |
| *ALG12* | *CUL4B* | *GNPTAB* | *NSD1* | *SLC35C1* |
| *ALMS1* | *CUL7* | *GPC3* | *OCRL* | *SLC35D1* |
| *ALPL* | *CYP11B1* | *GUSB* | *OFD1* | *SLC37A4* |
| *ANKH* | *CYP19A1* | *HCCS* | *PAPSS2* | *SLC39A13* |
| *ARL6* | *CYP21A2* | *HESX1* | *PAX3* | *SLC4A4* |
| *ARSB* | *CYP27B1* | *HMGA2* | *PAX8* | *SLC6A8* |
| *ARSE* | *DHCR7* | *HOXD13* | *PCNT* | *SMARCAL1* |
| *ATP6V0A2* | *DYM* | *HPRT1* | *PEX7* | *SMC1A* |
| *ATP7A* | *EBP* | *HRAS* | *PHEX* | *SMC3* |
| *ATP8B1* | *EFNB1* | *HSPG2* | *PHF6* | *SMPD1* |
| *ATR* | *EIF2AK3* | *HYAL1* | *PITX2* | *SMS* |
| *ATRX* | *ERCC2* | *ICK* | *POU1F1* | *SOS1* |
| *B3GALTL* | *ERCC3* | *IDUA* | *PQBP1* | *SOST* |
| *B4GALT7* | *ESCO2* | *IFT80* | *PROP1* | *SOX3* |
| *BBS1* | *EVC* | *IGBP1* | *PTCH1* | *SPG20* |
| *BBS10* | *EVC2* | *IGF1* | *PTCH2* | *SRY* |
| *BBS12* | *EXT1* | *IGF1R* | *PTEN* | *STAT5B* |
| *BBS2* | *EXT2* | *IGF2* | *PTH1R* | *TAZ* |
| *BBS4* | *FANCA* | *IHH* | *PTPN11* | *TBCE* |
| *BBS5* | *FANCB* | *IKBKG* | *RAB23* | *TBX1* |
| *BBS7* | *FANCC* | *JAG1* | *RAB3GAP1* | *TBX15* |
| *BBS9* | *FANCD2* | *KCNJ2* | *RAB3GAP2* | *TCF4* |
| *BMPR1B* | *FANCE* | *KDM5C* | *RAF1* | *TGFBR1* |
| *BRAF* | *FANCF* | *KIAA1279* | *RAI1* | *TGFBR2* |
| *BRCA2* | *FANCG* | *KRAS* | *RBM28* | *THRB* |
| *BTK* | *FANCI* | *LBR* | *RECQL4* | *TNFRSF11B* |
| *BUB1B* | *FANCL* | *LEMD3* | *RMRP* | *TP63* |
| *C7orf11* | *FANCM* | *LEPRE1* | *RNF135* | *TRAPPC2* |
| *CA2* | *FBN1* | *LHX4* | *ROR2* | *TRIM32* |
| *CCDC28B* | *FBN2* | *LIFR* | *RPL11* | *TRIM37* |
| *CEP290* | *FGD1* | *LIG4* | *RPL35A* | *TRPS1* |
| *CHD7* | *FGF23* | *LMNA* | *RPL5* | *TRPV4* |
| *CHRNG* | *FGFR2* | *LRP5* | *RPS17* | *UBR1* |
| *CHST3* | *FGFR3* | *MAP2K1* | *RPS19* | *WNT7A* |
| *CLCN5* | *FLNA* | *MAP2K2* | *RPS24* | *WRN* |
| *COL10A1* | *FLNB* | *MATN3* | *RPS6KA3* | *ZBTB16* |
| *COL11A1* | *FOXC1* | *MC4R* | *RPS7* |  |
| *COL11A2* | *FUCA1* | *MECP2* | *RUNX2* |  |
| *COL1A1* | *G6PC* | *MGP* | *SBDS* |  |
| *COL1A2* | *GALNS* | *MKKS* | *SDHA* |  |
| *COL2A1* | *GDF5* | *MKS1* | *SECISBP2* |  |
| *COL5A1* | *GH1* | *MMP13* | *SEMA3E* |  |
| *COL5A2* | *GHR* | *MRPS16* | *SHH* |  |
| *COL9A1* | *GHRHR* | *MYCN* | *SHOX* |  |
| *COL9A2* | *GHSR* | *NBN* | *SHROOM4* |  |

Table 4. The variants found in other genes related to short stature.

| Variation  Location | Gene(s) | Protein change | Condition(s) | Clinical significance |
| --- | --- | --- | --- | --- |
| [NM_020765.3(UBR4):c.13828C>A (p.Pro4610Thr)](https://www.ncbi.nlm.nih.gov/clinvar/variation/599553/)  GRCh37:  Chr1:19420552  GRCh38:  Chr1:19094058 | *[UBR4](https://www.ncbi.nlm.nih.gov/gene/23352)* | P4610T | Short stature | Likely pathogenic |
| [NM_020765.3(UBR4):c.10388G>A (p.Arg3463His)](https://www.ncbi.nlm.nih.gov/clinvar/variation/599550/)  GRCh37:  Chr1:19446118  GRCh38:  Chr1:19119624 | *[LOC126805641](https://www.ncbi.nlm.nih.gov/gene/126805641), [UBR4](https://www.ncbi.nlm.nih.gov/gene/23352)* | R3463H | Short stature | Likely pathogenic |
| [NM_020765.3(UBR4):c.7031A>G (p.Asn2344Ser)](https://www.ncbi.nlm.nih.gov/clinvar/variation/599501/)  GRCh37:  Chr1:19478319  GRCh38:  Chr1:19151825 | *[UBR4](https://www.ncbi.nlm.nih.gov/gene/23352)* | N2344S | Short stature | Likely pathogenic |
| [NM_020765.3(UBR4):c.6325G>A (p.Gly2109Ser)](https://www.ncbi.nlm.nih.gov/clinvar/variation/599552/)  GRCh37:  Chr1:19481545  GRCh38:  Chr1:19155051 | *[UBR4](https://www.ncbi.nlm.nih.gov/gene/23352)* | G2109S | Short stature | Likely pathogenic |
| [NM_013943.3(CLIC4):c.220C>T (p.His74Tyr)](https://www.ncbi.nlm.nih.gov/clinvar/variation/599503/)  GRCh37:  Chr1:25140622  GRCh38:  Chr1:24814131 | *[CLIC4](https://www.ncbi.nlm.nih.gov/gene/25932)* | H74Y | Short stature | Likely pathogenic |
| [NM_014874.4(MFN2):c.280C>T (p.Arg94Trp)](https://www.ncbi.nlm.nih.gov/clinvar/variation/2276/)  GRCh37:  Chr1:12052716  GRCh38:  Chr1:11992659 | *[MFN2](https://www.ncbi.nlm.nih.gov/gene/9927)* | R94W | Inborn genetic diseases, Charcot-Marie-Tooth disease, Charcot-Marie-Tooth disease type 2, not provided, Hereditary motor and sensory neuropathy with optic atrophy,  Short stature, Failure to thrive | Pathogenic |
| [NM_133178.4(PTPRU):c.2531C>T (p.Pro844Leu)](https://www.ncbi.nlm.nih.gov/clinvar/variation/599583/)  GRCh37:  Chr1:29630421  GRCh38:  Chr1:29303909 | *[PTPRU](https://www.ncbi.nlm.nih.gov/gene/10076)* | P844L, P854L | Short stature | Likely pathogenic |
| [NM_133178.4(PTPRU):c.3136G>A (p.Val1046Ile)](https://www.ncbi.nlm.nih.gov/clinvar/variation/599585/)  GRCh37:  Chr1:29639127  GRCh38:  Chr1:29312615 | *[PTPRU](https://www.ncbi.nlm.nih.gov/gene/10076)* | V1043I, V1056I, V1046I, V1052I | Short stature | Uncertain significance |
| [NM_133178.4(PTPRU):c.4165A>C (p.Met1389Leu)](https://www.ncbi.nlm.nih.gov/clinvar/variation/599584/)  GRCh37:  Chr1:29651755  GRCh38:  Chr1:29325243 | *[LOC122056818](https://www.ncbi.nlm.nih.gov/gene/122056818), [PTPRU](https://www.ncbi.nlm.nih.gov/gene/10076)* | M1386L, M1399L, M1389L, M1393L | Short stature | Likely pathogenic |
| [NM_001394062.1(MACF1):c.3827G>A (p.Arg1276Gln)](https://www.ncbi.nlm.nih.gov/clinvar/variation/599522/)  GRCh37:  Chr1:39784169  GRCh38:  Chr1:39318497 | *[MACF1](https://www.ncbi.nlm.nih.gov/gene/23499)* | R1281Q, R1276Q | Short stature | Likely pathogenic |
| [NM_001394062.1(MACF1):c.12482A>G (p.Lys4161Arg)](https://www.ncbi.nlm.nih.gov/clinvar/variation/599521/)  GRCh37:  Chr1:39827060  GRCh38:  Chr1:39361388 | *[MACF1](https://www.ncbi.nlm.nih.gov/gene/23499)* | K2099R, K4161R | Short stature | Uncertain significance |
| [NM_001394062.1(MACF1):c.17989-11C>T](https://www.ncbi.nlm.nih.gov/clinvar/variation/599524/)  GRCh37:  Chr1:39903438  GRCh38:  Chr1:39437766 | *[MACF1](https://www.ncbi.nlm.nih.gov/gene/23499)* |  | Short stature | Uncertain significance |
| [NM_001394062.1(MACF1):c.18687G>T (p.Trp6229Cys)](https://www.ncbi.nlm.nih.gov/clinvar/variation/599525/)  GRCh37:  Chr1:39907638  GRCh38:  Chr1:39441966 | *[MACF1](https://www.ncbi.nlm.nih.gov/gene/23499)* | W4170C, W6229C | Short stature | Uncertain significance |
| [NM_001394062.1(MACF1):c.22433G>A (p.Arg7478His)](https://www.ncbi.nlm.nih.gov/clinvar/variation/599523/)  GRCh37:  Chr1:39951231  GRCh38:  Chr1:39485559 | *[MACF1](https://www.ncbi.nlm.nih.gov/gene/23499)* | R5353H, R7478H | Short stature | Likely pathogenic |
| [NM_001384.5(DPH2):c.224C>G (p.Ser75Ter)](https://www.ncbi.nlm.nih.gov/clinvar/variation/2671662/)  GRCh37:  Chr1:44436344  GRCh38:  Chr1:43970672 | *[DPH2](https://www.ncbi.nlm.nih.gov/gene/1802)* | Q15E, S75* | Developmental delay with short stature, dysmorphic facial features, and sparse hair 2 | Likely pathogenic |
| [NM_001384.5(DPH2):c.601C>T (p.Arg201Cys)](https://www.ncbi.nlm.nih.gov/clinvar/variation/872919/)  GRCh37:  Chr1:44437175  GRCh38:  Chr1:43971503 | *[DPH2](https://www.ncbi.nlm.nih.gov/gene/1802), [LOC126805726](https://www.ncbi.nlm.nih.gov/gene/126805726)* | R125C, R201C, R53C, R66C, R8C | Developmental delay with short stature, dysmorphic facial features, and sparse hair 2, Short stature, Global developmental delay, Ventricular septal defect | Conflicting interpretations of pathogenicity |
| [NM_001384.5(DPH2):c.922C>T (p.Gln308Ter)](https://www.ncbi.nlm.nih.gov/clinvar/variation/872918/)  GRCh37:  Chr1:44437496  GRCh38:  Chr1:43971824 | *[DPH2](https://www.ncbi.nlm.nih.gov/gene/1802), [LOC126805726](https://www.ncbi.nlm.nih.gov/gene/126805726)* | Q115*, Q160*, Q173*, Q217*, Q232*, Q308* | Developmental delay with short stature, dysmorphic facial features, and sparse hair 2, Short stature, Global developmental delay, Ventricular septal defect | Conflicting interpretations of pathogenicity |
| [NM_015306.3(USP24):c.7448-7C>T](https://www.ncbi.nlm.nih.gov/clinvar/variation/599595/)  GRCh37:  Chr1:55539586  GRCh38:  Chr1:55073913 | *[USP24](https://www.ncbi.nlm.nih.gov/gene/23358)* |  | Short stature | Likely pathogenic |
| [NM_015306.3(USP24):c.2747A>T (p.Tyr916Phe)](https://www.ncbi.nlm.nih.gov/clinvar/variation/599596/)  GRCh37:  Chr1:55607292  GRCh38:  Chr1:55141619 | *[USP24](https://www.ncbi.nlm.nih.gov/gene/23358)* | Y916F | Short stature | Uncertain significance |
| [NM_015306.3(USP24):c.2491A>G (p.Met831Val)](https://www.ncbi.nlm.nih.gov/clinvar/variation/599597/)  GRCh37:  Chr1:55608741  GRCh38:  Chr1:55143068 | *[USP24](https://www.ncbi.nlm.nih.gov/gene/23358)* | M831V | Short stature | Uncertain significance |
| [NM_015958.3(DPH5):c.779A>G (p.His260Arg)](https://www.ncbi.nlm.nih.gov/clinvar/variation/1708532/)  GRCh37:  Chr1:101456043  GRCh38:  Chr1:100990487 | *[DPH5](https://www.ncbi.nlm.nih.gov/gene/51611), [SLC30A7](https://www.ncbi.nlm.nih.gov/gene/148867)* | H260R, C13R, H259R | Neurodevelopmental disorder with short stature, prominent forehead, and feeding difficulties | Pathogenic |
| [NM_015958.3(DPH5):c.619C>T (p.Arg207Ter)](https://www.ncbi.nlm.nih.gov/clinvar/variation/1708533/)  GRCh37:  Chr1:101458208  GRCh38:  Chr1:100992652 | *[SLC30A7](https://www.ncbi.nlm.nih.gov/gene/148867), [DPH5](https://www.ncbi.nlm.nih.gov/gene/51611)* |  | Neurodevelopmental disorder with short stature, prominent forehead, and feeding difficulties | Pathogenic |
| [NM_032305.3(POLR3GL):c.326-1G>A](https://www.ncbi.nlm.nih.gov/clinvar/variation/617636/)  GRCh37:  Chr1:145457605  GRCh38:  Chr1:145977482 | *[LOC129931343](https://www.ncbi.nlm.nih.gov/gene/129931343), [POLR3GL](https://www.ncbi.nlm.nih.gov/gene/84265)* |  | Short stature, oligodontia, dysmorphic facies, and motor delay, Abnormal facial shape, Short stature, Hyperostosis, Oligodontia | Pathogenic/Likely pathogenic |
| [NM_004284.6(CHD1L):c.1929del (p.Arg643fs)](https://www.ncbi.nlm.nih.gov/clinvar/variation/599533/)  GRCh37:  Chr1:146757075  GRCh38:  Chr1:147285398 | *[CHD1L](https://www.ncbi.nlm.nih.gov/gene/9557)* | R480fs, R491fs, R543fs, R571fs, R439fs, R530fs, R362fs, R643fs | Short stature | Pathogenic |
| [NM_015100.4(POGZ):c.402_409dup (p.His137fs)](https://www.ncbi.nlm.nih.gov/clinvar/variation/373957/)  GRCh37:  Chr1:151403191-151403192  GRCh38:  Chr1:151430715-151430716 | *[POGZ](https://www.ncbi.nlm.nih.gov/gene/23126)* | H137fs, H84fs | Short metacarpal, Myopia, Intellectual disability, Truncal obesity, Hypothyroidism, Hearing impairment, Short stature | Likely pathogenic |
| [NM_014856.3(DENND4B):c.2828G>C (p.Trp943Ser)](https://www.ncbi.nlm.nih.gov/clinvar/variation/599506/)  GRCh37:  Chr1:153906724  GRCh38:  Chr1:153934248 | *[DENND4B](https://www.ncbi.nlm.nih.gov/gene/9909)* | W943S,W954S | Short stature | Uncertain significance |
| [NM_014856.3(DENND4B):c.1825C>T (p.Leu609Phe)](https://www.ncbi.nlm.nih.gov/clinvar/variation/599505/)  GRCh37:  Chr1:153911516  GRCh38:  Chr1:153939040 | *[DENND4B](https://www.ncbi.nlm.nih.gov/gene/9909)* | L609F, L620F | Short stature | Likely pathogenic |
| [NM_006912.6(RIT1):c.67A>C (p.Lys23Gln)](https://www.ncbi.nlm.nih.gov/clinvar/variation/224122/)  GRCh37:  Chr1:155880486  GRCh38:  Chr1:155910695 | *[RIT1](https://www.ncbi.nlm.nih.gov/gene/6016)* | K23Q, K40Q | Pedal edema, Short stature, Hypertelorism, Downslanted palpebral fissures, not provided, Noonan syndrome 8, not specified, Cardiovascular phenotype | Conflicting interpretations of pathogenicity |
| [NM_002107.7(H3-3A):c.386G>A (p.Arg129His)](https://www.ncbi.nlm.nih.gov/clinvar/variation/1183981/)  GRCh37:  Chr1:226259155  GRCh38:  Chr1:226071454 | *[H3-3A](https://www.ncbi.nlm.nih.gov/gene/3020)* | R129H | Bryant-Li-Bhoj neurodevelopmental syndrome 1, Intellectual disability, Delayed speech and language development, Short stature, Brain imaging abnormality, Global developmental delay | Pathogenic/Likely |
| [NM_002107.7(H3-3A):c.121C>T (p.Arg41Cys)](https://www.ncbi.nlm.nih.gov/clinvar/variation/1183977/)  GRCh37:  Chr1:226252173  GRCh38:  Chr1:226064472 | *[H3-3A](https://www.ncbi.nlm.nih.gov/gene/3020)* | R41C | Bryant-Li-Bhoj neurodevelopmental syndrome 1, Intellectual disability, Delayed speech and language development, Short stature, Brain imaging abnormality, Global developmental delay | Pathogenic/Likely pathogenic |
| [NM_018060.4(IARS2):c.2726C>T (p.Pro909Leu)](https://www.ncbi.nlm.nih.gov/clinvar/variation/156553/)  GRCh37:  Chr1:220316451  GRCh38:  Chr1:220143109 | *[IARS2](https://www.ncbi.nlm.nih.gov/gene/55699)* | P909L | Cataract-growth hormone deficiency-sensory neuropathy-sensorineural hearing loss-skeletal dysplasia syndrome, Peripheral neuropathy, growth hormone deficiency with short stature, Cataract, partial sensorineural deafness | Pathogenic |
| [NM_032833.5(PPP1R15B):c.1972C>T (p.Arg658Cys)](https://www.ncbi.nlm.nih.gov/clinvar/variation/222030/)  GRCh37:  Chr1:204375390  GRCh38:  Chr1:204406262 | *[PPP1R15B](https://www.ncbi.nlm.nih.gov/gene/84919)* | R658C | Microcephaly, short stature, and impaired glucose metabolism 2 | Pathogenic |
| [NM_145697.3(NUF2):c.371T>G (p.Ile124Ser)](https://www.ncbi.nlm.nih.gov/clinvar/variation/992629/)  GRCh37:  Chr1:163306574  GRCh38:  Chr1:163336784 | *[NUF2](https://www.ncbi.nlm.nih.gov/gene/83540)* | I124S | Short stature, Microcephaly | Pathogenic |

For more information, please visit the website: <https://maayanlab.cloud/Harmonizome/resource/ClinVar>
